# Intelligent Grading of Green Cardamom Using Data Fusion of Electronic Nose and Computer Vision Methods

**DOI:** 10.1002/fsn3.4645

**Published:** 2025-03-27

**Authors:** Ehsan Godini, Hemad Zareiforoush, Adel Bakhshipour, Zahra Lorigooini, Sayed Hossain Payman

**Affiliations:** ^1^ Department of Biosystems Engineering, Faculty of Agricultural Sciences University of Guilan Rasht Iran; ^2^ Medical Plants Research Center, Basic Health Sciences Institute Shahrekord University of Medical Sciences Shahrekord Iran

**Keywords:** aromatic spices, data mining, image processing, non‐destructive test, quality

## Abstract

In this research, the intelligent quality grading of green cardamom was carried out using electronic nose (e‐nose) and computer vision (CV) methods along with machine learning (ML) approaches. Cardamom samples were analyzed in three grades including Grade 1 (healthy and green), Grade 2 (healthy with yellow color), and Grade 3 (immature and shriveled) for capsules and Grade 1 (Black), Grade 2 (Brown), and Grade 3 (Yellow and red) for seeds. Three ML algorithms including Decision Tree (DT), Bayesian Network (BN), and Support Vector Machine (SVM) were used to classify the quality grades. Results showed that the correlation‐based feature selection (CFS) algorithm decreased the number of input features and increased the classification performance. For classifying cardamom capsule samples based on the visual features, the CFS‐BN model was the best classifier, with the root mean squared error (RMSE) and accuracy of 0.1408 and 96.67%, respectively. The RMSE and accuracy of this model for classifying cardamom seeds based on image features were 0.1220 and 96.67%, respectively. In classifying cardamom seeds using e‐nose data, the CFS‐DT model was the best classifier with RMSE and accuracy of 0.2093 and 93.33%, respectively. The CFS‐BN model was the best for classifying cardamom capsules with an RMSE of 0.1126 and an accuracy of 96.67%. The fusion of e‐nose and CV data increased the model performance compared to the separate use of e‐nose and CV datasets. The accuracy of the CFS‐BN model using the combination of CV and e‐nose data was 100% during both the calibration and evaluation stages. It can be concluded that data fusion of e‐nose and CV methods can be effectively used to develop an intelligent, accurate, reliable, fast, and non‐destructive system for quality grading of cardamom capsules and seeds.

## Graphical abstract

In this research, the intelligent quality grading of green cardamom was carried out using electronic nose (e‐nose) and computer vision (CV) methods along with machine learning (ML) approaches. The results of the classifiers' performance evaluation based on the data fusion method, compared to the separate use of features obtained from CV and e‐nose systems, clearly showed that combining e‐nose sensors information with CV can significantly improve the detection capability and confidence in the quality grading of cardamom capsules and seeds. The accuracy of the combined system with the application of the CFS‐BN model in cardamom product grading was 100% in both the calibration and evaluation stages. This study provides valuable insights for the development of a fast, accurate, and non‐destructive system based on the fusion of CV and e‐nose data for quality the grading of cardamom products.

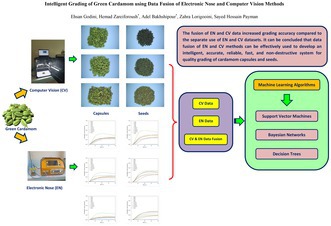

## 1 Introduction

Green cardamom is one of the most important medicinal plants, ranked third in the world in terms of price and economic value among aromatic spices, after saffron and vanilla. Cardamom is mainly cultivated in Guatemala, Indonesia, India, and Nepal. This product is also found in Tanzania, Indonesia, Vietnam, Thailand, Papua New Guinea, and El Salvador and is exported to all over the world, especially to Middle Eastern countries (FAOSTAT 2022). Cardamom essential oil is used as a food flavoring and has a wide range of potential therapeutic applications (Ghosh et al. 2022). El Malti, Mountassif, and Amarouch (2007) demonstrated that green cardamom essential oil has antimicrobial activity against pathogenic strains, highlighting its value as a spice worldwide. This powerful antioxidant plant has a historical record dating back to 3000 BC. Known as the queen of spices, it contains major metabolites such as alpha‐pinene, beta‐pinene, sabinene, myrcene, alpha‐phellandrene, limonene, cineole, terpinen, cymene, terpinenol, linalool, and other various biological components that cause anti‐cancer, anti‐inflammatory, anti‐fungal, anti‐bacterial, and antioxidant properties (Abu‐Taweel 2018). Depending on the product's conditions during the growth and processing period, different quality grades of cardamom are offered in the market in the form of whole capsules or seeds, which have different prices. The grading of cardamom quality in commercial markets is based on various factors according to international standards. In addition to physical characteristics such as color, shape, and size, the ripeness, hollowness, deformity, shriveled skin, moldiness, and the amount of aroma are also considered important and fundamental criteria for cardamom quality assessment (ISO 2018). Currently, the grading of cardamom capsules and seeds on a commercial scale is mainly carried out using manual and mechanical methods, which lack adequate accuracy.

Manual inspection of the cardamom product based solely on its external characteristics is usually used to determine the product quality. However, in some cases, cardamom capsule samples with acceptable appearance contain low‐quality seeds. The measurement of cardamom seed quality indicators such as aroma and flavor is primarily based on essential oil extraction and its chemical analysis using laboratory methods like gas chromatography–mass spectrometry (GC–MS). The time‐consuming, expensive, and destructive nature of these traditional and conventional methods necessitates the design of new non‐destructive quality assessment systems for implementation in the cardamom packaging and processing industry. Studies have demonstrated the potential of employing novel measurement techniques such as electronic nose (e‐nose) and computer vision (CV) in conjunction with machine learning (ML) algorithms for non‐destructive quality assessment of aromatic medicinal plants like cardamom. CV is a fast and accurate technique that has demonstrated successful performance in various agricultural and food industries, including the monitoring and grading of fruits, vegetables, and medicinal plants (Wan et al. 2018). In recent years, researchers have explored the potential of CV and image processing techniques for quality assessment of agricultural and food products (Gomes and Leta 2012; Kodagali and Balaji 2012; Sapna and Sheshappa 2022; Tian et al. 2020). Sarkar et al. (2023) investigated the discrimination of red chili powder from brick powder using color space filters and ML algorithms (RGB, HSV, Lab, and YCbCr). They reported satisfactory performance of the CV approach.

In a study, an image processing technique was proposed to classify rice quality levels using color and textural features. Seven categories of paddy grains were imaged and analyzed. The highest classification accuracy (93.31%) was achieved by a multilayer perceptron (MLP) back‐propagation neural network (BPNN) classifier in combination with principal component analysis (PCA) method (Anami et al. 2019). In another study on soybean classification using an effective image‐based learning approach, the performance of the CV system was reported to be 82% accurate after processing hundreds of soybean images (Souza Jr et al. 2024).

E‐nose, also known as an artificial olfactory system, is a device that mimics the human sense of smell (Sun et al. 2019). E‐noses are instruments based on an array of gas sensors designed to detect and discriminate between aromatic compounds and complex odors (Santos et al. 2019). Volatile organic compounds (VOCs) released by food products can be captured and identified using e‐nose systems. These VOCs provide valuable information about the health status of food products (Effah 2020). The application of e‐nose in quality assessment of agricultural and food products has been extensively investigated by researchers in recent years (Ali et al. 2020; Jia et al. 2019; Loutfi et al. 2015).

In a study, an e‐nose system with eight metal oxide semiconductor (MOS) sensors was developed for the discrimination of green cumin seeds using chemometrics tools. The system achieved 100% accuracy by the two‐dimensional linear discriminant analysis (2D‐LDA) algorithm and 87.5% accuracy by partial least squares discriminant analysis (PLS‐DA; Ghasemi‐Varnamkhasti, Amiri, et al. 2018a). In another study, an e‐nose system based on MOS sensors was designed and utilized for the classification of essential oil composition in different genotypes of rose flowers by employing PCA, LDA, and SVM classification models. The highest accuracy of 99% was achieved using the SVM model (Gorji‐Chakespari et al. 2017). In another study, the combination of an e‐nose and ML techniques was employed to determine the changes in the aroma of tarragon essential oil based on temperature and airflow rate variations in a hybrid dryer with 100% accuracy (Karami et al. 2024). Moreover, the quality classification of chili powder was successfully performed using the e‐nose sensor array and ML algorithms of support vector machines (SVMs), decision tree (DT), and random forest (RF) models (Ma et al. 2022).

One of the interesting fields of ML in recent years has been the fusion of data from different non‐destructive sources in order to increase the performance of food evaluation studies. Data fusion, also known as data integration or data merging, is a novel approach in the field of food quality assessment that combines data obtained from various quality evaluation methods to achieve the most comprehensive information about the product's quality status and maximize the accuracy of quality grading. In a study, the fusion of near‐infrared spectroscopy (NIRS) and machine vision data was used for rapid identification of impure rice grains. The classifying models included SVM, RF, and gradient boosting tree (GBT). The results demonstrated that the classification performance was significantly improved compared to using NIRS and machine vision data individually. Among the evaluated models, the GBT‐based model achieved the highest classification accuracy with 100% recognition rates (Song et al. 2024). In another study on the classification of pollen collected by honey bees based on the origin of the collection, three methods of e‐nose, electronic tongue, and spectroscopy were used. It was reported that data fusion of the three methods, with a 100% classification accuracy, resulted in superior performance compared to the separate models (Sipos et al. 2020).

Due to the high economic value of green cardamom as a globally traded commodity and its medicinal properties with numerous health benefits, it is important to use intelligent and ML‐based quality evaluation methods in processing industries to enhance the grading speed and accuracy. A review of the existing scientific literature reveals that no previous study has investigated the quality evaluation of green cardamom using data fusion of aroma and image‐based features together with ML techniques. Therefore, this study aimed to perform data fusion of CV and e‐nose systems using metaheuristic algorithms for the quality classification of green cardamom capsules and seeds.

## 2 Materials and Methods

### 2.1 Sample Preparation

The experiments were conducted in August 2023 in the Postharvest Technology Laboratory of the Biosystems Engineering Department, Faculty of Agricultural Sciences, University of Guilan, Rasht, Iran. Two kilograms of cured capsules of green cardamom was purchased from a reputable herbal medicine store in Rasht city. Based on the definitions provided by international standards, cardamom capsule samples were classified into three quality grades (ISO 2018). The grades included Grade 1 (healthy and green capsules), Grade 2 (healthy capsules with yellow color), and Grade 3 (immature and shriveled capsules). Additionally, the cardamom seeds were graded into three quality grades: Grade 1 (Black seeds), Grade 2 (Brown seeds), and Grade 3 (Yellow and red seeds). Fifteen samples weighing 30 g were prepared for each quality grade of cardamom capsules and seeds. The samples were placed in polypropylene zipper bags and stored at room temperature.

### 2.2 E‐Nose System

**FIGURE 1:**
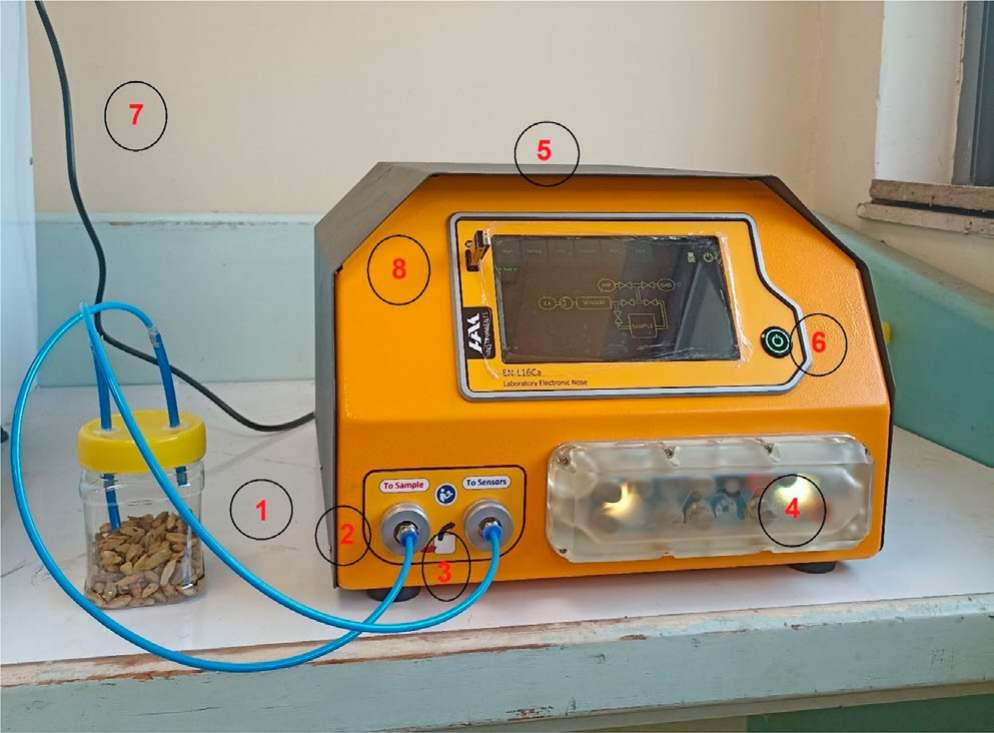
The e‐nose system used in this study: (1) sample container, (2) inlet air from the air filter, (3) sample aroma transmission pipe to the sensors housing, (4) sensors housing, (5) chamber containing solenoid valves and electronic board, (6) graphical user interface, (7) power supply cable, and (8) data storage unit.

To obtain information from the aroma of cardamom samples, a laboratory e‐nose system was employed. The system consisted of a sample container, gas sensors, an air pump, and solenoid valves for controlling the airflow, a carbon air filter, an electronic board for data collection, a graphical display, and sensor housing (Figure 1).

In this study, nine MOS sensors were used, the specifications of which are presented in Table 1. Two carbon filters were utilized in the e‐nose system in the path of ambient air to the sensor chamber and sample container to remove any contaminants and purify the air entering the system.

**TABLE 1:** General characters of sensors working in the e‐nose system.

Number of sensors	Sensor name	Reaction material	Limits of detection (ppm)
1	MQ2	LPG, I‐butane, propane, methane, alcohol, H2, smoke	300‐5000‐LPG and propane, 5000‐butane, 20,000‐methane, 300‐5000‐H2, 100–2000 Alcohol
2	MQ4	CH4, CNG	300–10,000
3	MQ6	LPG, C4H10, CH4, C3H8	300–10,000
4	MQ7	CO	100–300
5	MQ9	CO and CH4, LPG	20–2000 CO, 500–10,000 CH4, 500–10,000 LPG
6	TGS813	C4H10, CH4, C3H8, CO, NH3, SO2, C2H5OH, and C8H18	500–10,000
7	TGS822	Breath alcohol detectors, gas leak detectors/alarm, solvent detectors for factories, drycleaners, and semiconductor	50–5000
8	TGS2610	Butane, liquid petroleum gas	500–1000
9	TGS2611	CH4, Natural gas	500–10,000

**FIGURE 2:**
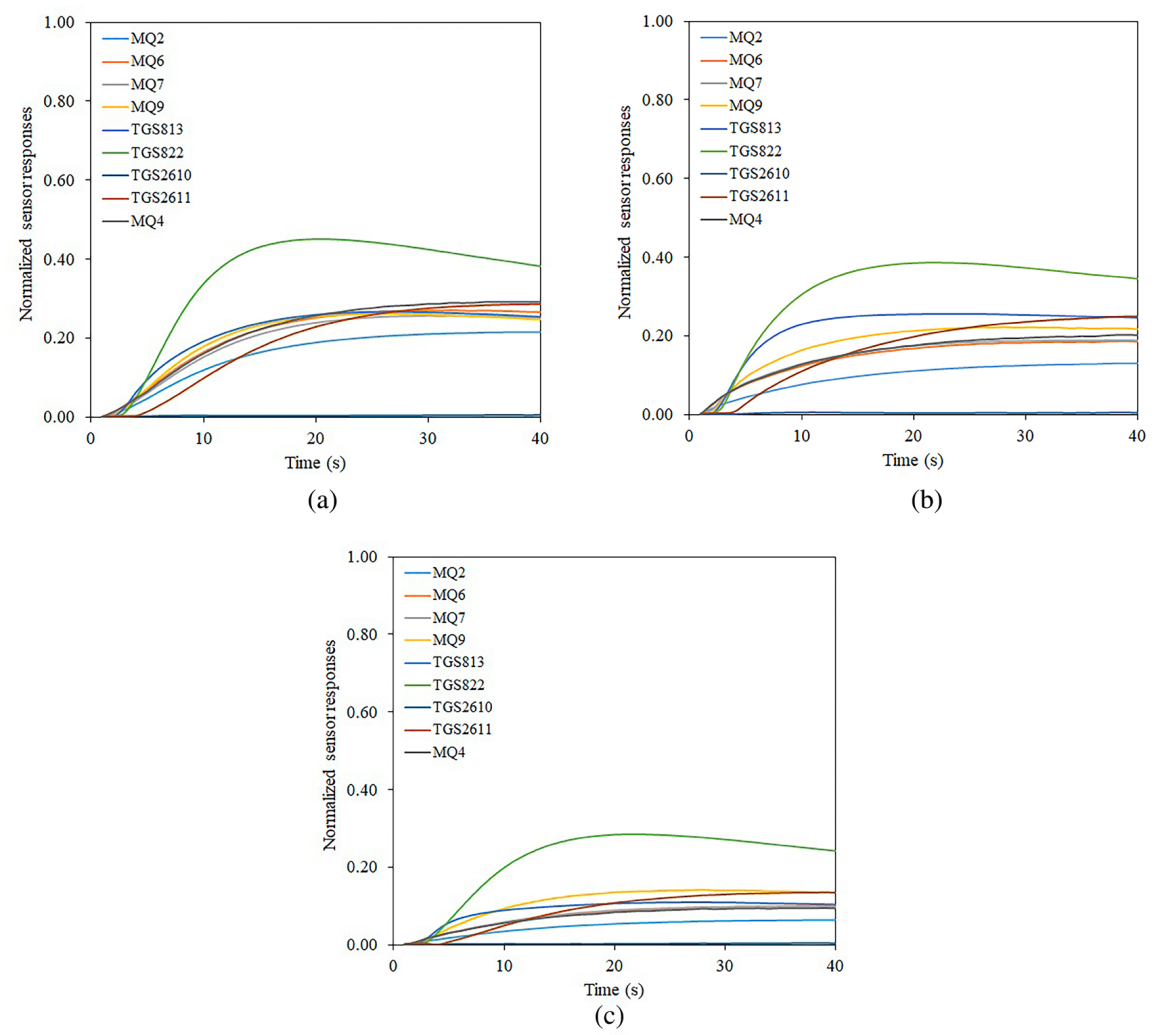
Sample preprocessed responses of the sensor array for different quality grades of cardamom capsules, (a) Grade 1, (b) Grade 2, and (c) Grade 3.

**FIGURE 3:**
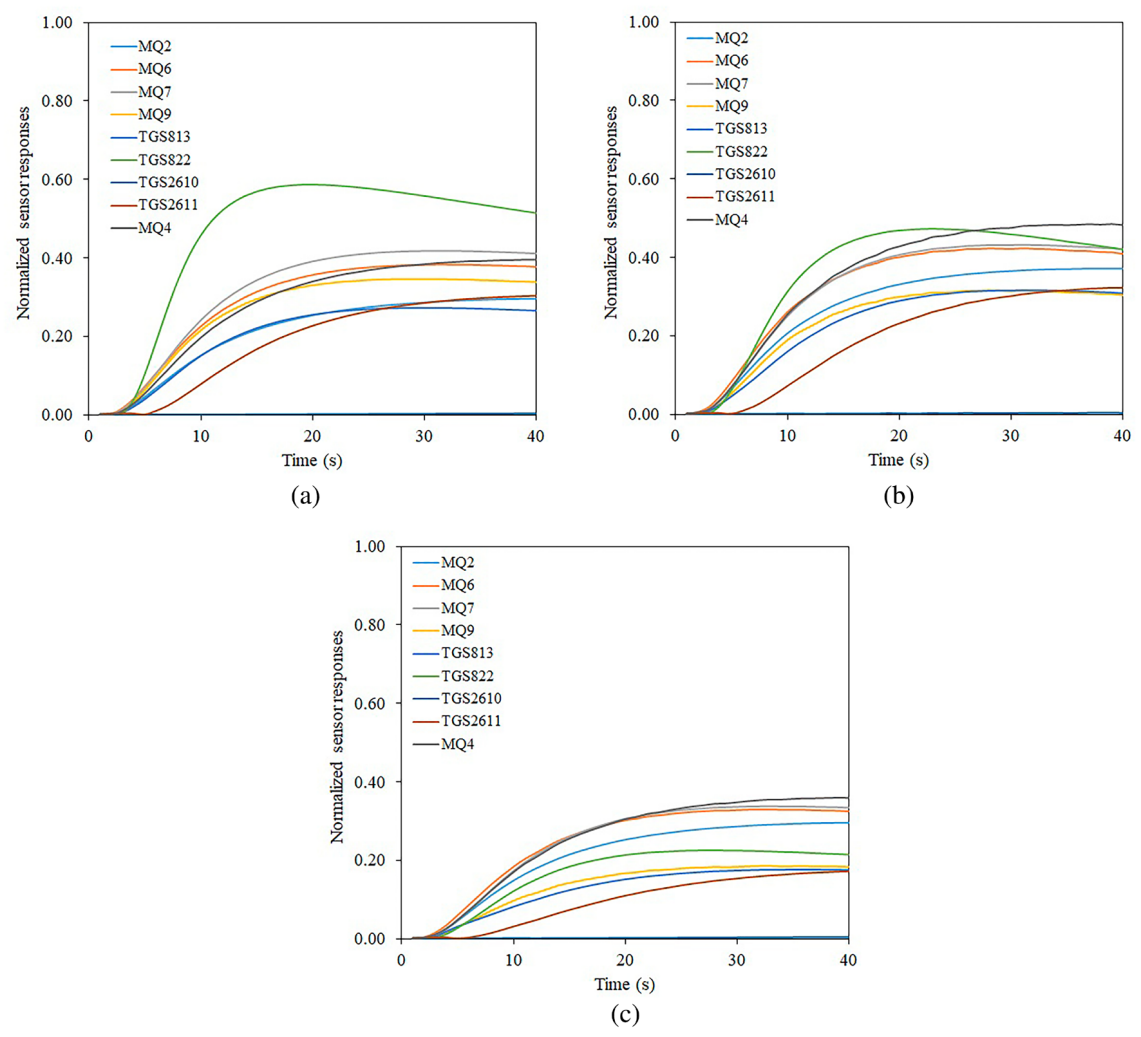
Sample preprocessed responses of the sensor array for different quality grades of cardamom seeds, (a) Grade 1, (b) Grade 2, and (c) Grade 3.

In order to perform the e‐nose experiments, 30 g samples of the cardamom product were placed in 200 mL sealed plastic containers and held in the container for 10 min to allow the headspace of the container to become saturated with the aroma of the cardamom product. The main operational steps of the e‐nose system were baseline correction (200 s), sample headspace injection (40 s), and sensor recovery (60 s). The duration of each phase was chosen based on pre‐experiments. During the baseline correction phase, purified ambient air was pumped into the sensors chamber to bring the sensors to a steady state. After baseline correction, the injection phase began, in which the odor from the samples headspace was pumped into the sensor array, resulting in a change in the output voltage of each sensor proportional to the chemical compounds present in the sample odor. In the final phase, purified ambient air was passed over the sensors to remove any remaining odor from the sensor chamber and prepare the system to collect data from the next sample. In order to extract the e‐nose features, the recorded e‐nose signals were called in the MATLAB programming software. Initially, the raw data were mathematically corrected and normalized using a fractional method (Ayari et al. 2018). In this study, the recorded and preprocessed data from the headspace of the sample (time range of 201–240 s) were selected for feature extraction. Figures 2 and 3 show the preprocessed response of the sensors used in the e‐nose system to different quality grades of cardamom capsule and seed samples, respectively. The difference in sensor responses to different classes of capsule and seed samples is clearly visible in these figures. Such distinction makes it possible to distinguish between different samples of cardamom and classify the product quality.

The features extracted from the preprocessed responses of the e‐nose system sensors included (Nimsuk 2019; Sanaeifar et al. 2021; Yin et al. 2014):

1. Maximum Sensor Response (MSR)
2. Integral value (INV), which is the area of the region under the response curve, representing the overall response of a sensor to a cardamom sample over a time interval.
3. Impregnation time (*T*_im_), which is defined as the time to reach the maximum response of a sensor.
4. Average slope of ascending section (*S*_asce_)
5. Maximum value of the instantaneous slopes (*S*_max_)
6. Mean differential coefficient value (MDCV), which represents the average response rate of the sensors in the selected range.

### 2.3 CV System

**FIGURE 4:**
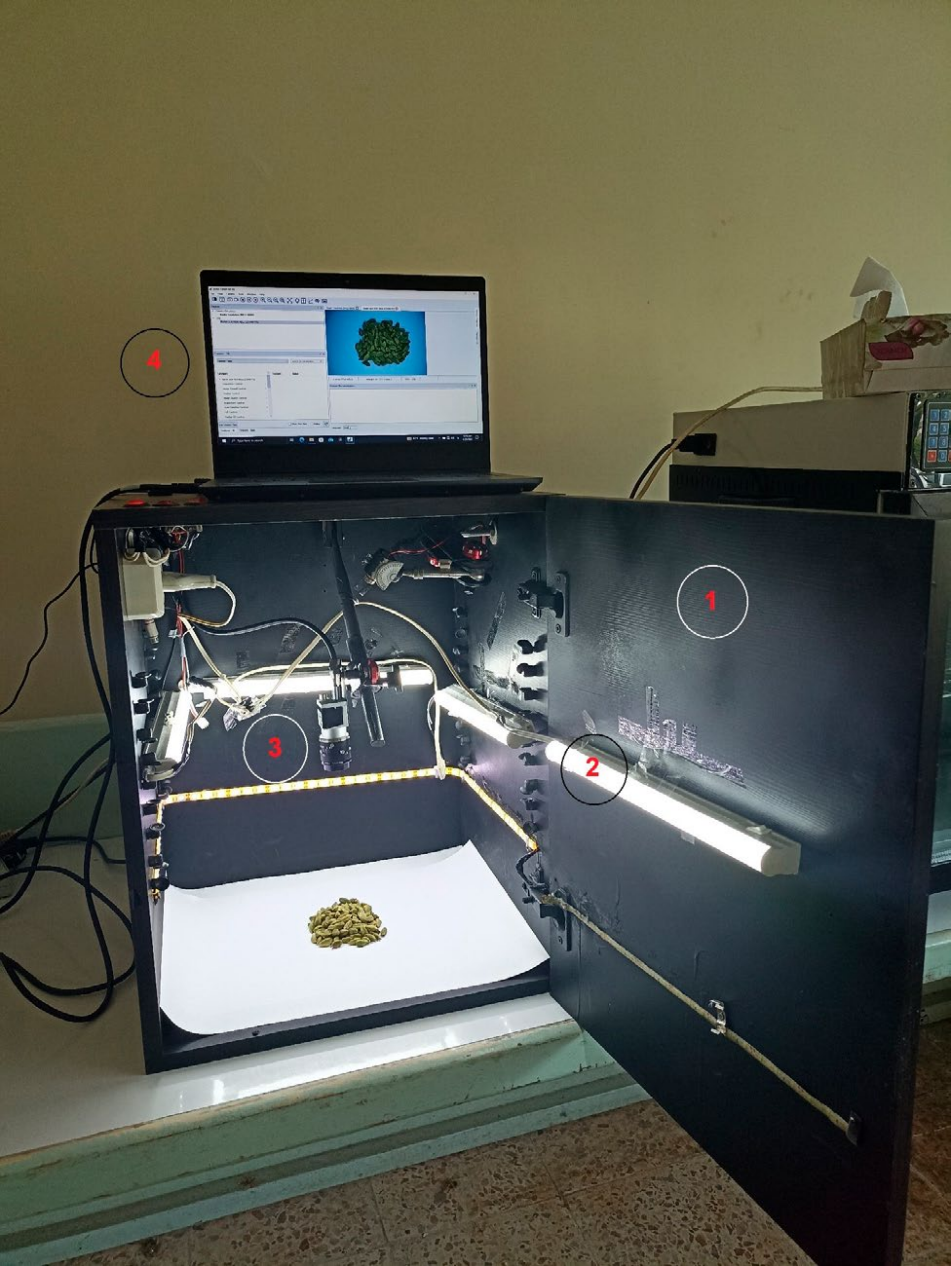
The setup of the CV system used to acquire the images of cardamom samples: (1) Imaging chamber, (2) fluorescent lamps, (3) digital camera, and (4) computer and user interface.

**FIGURE 5:**
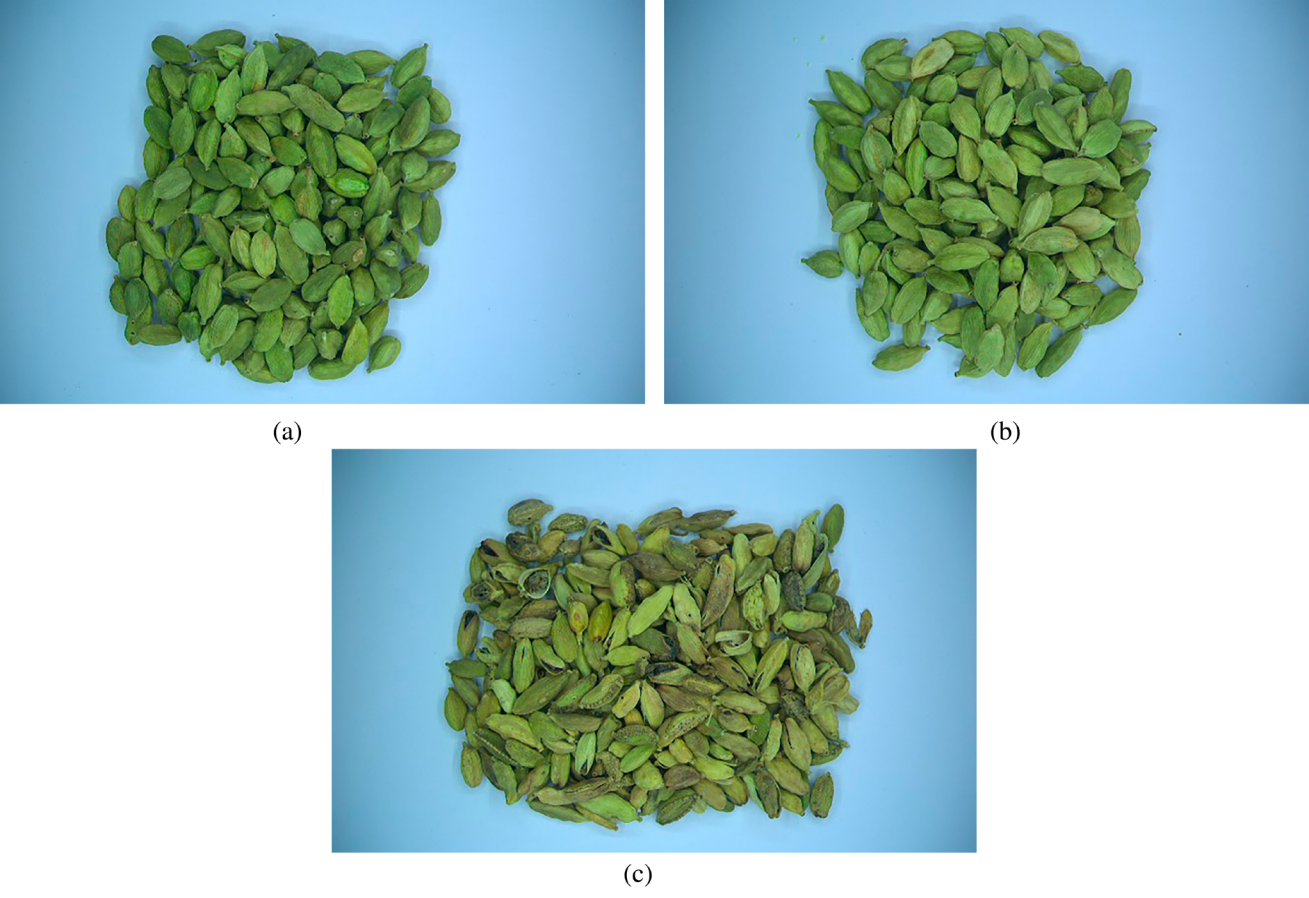
Images of different quality grades of cardamom capsules, (a) Grade 1, (b) Grade 2, and (c) Grade 3.

**FIGURE 6:**
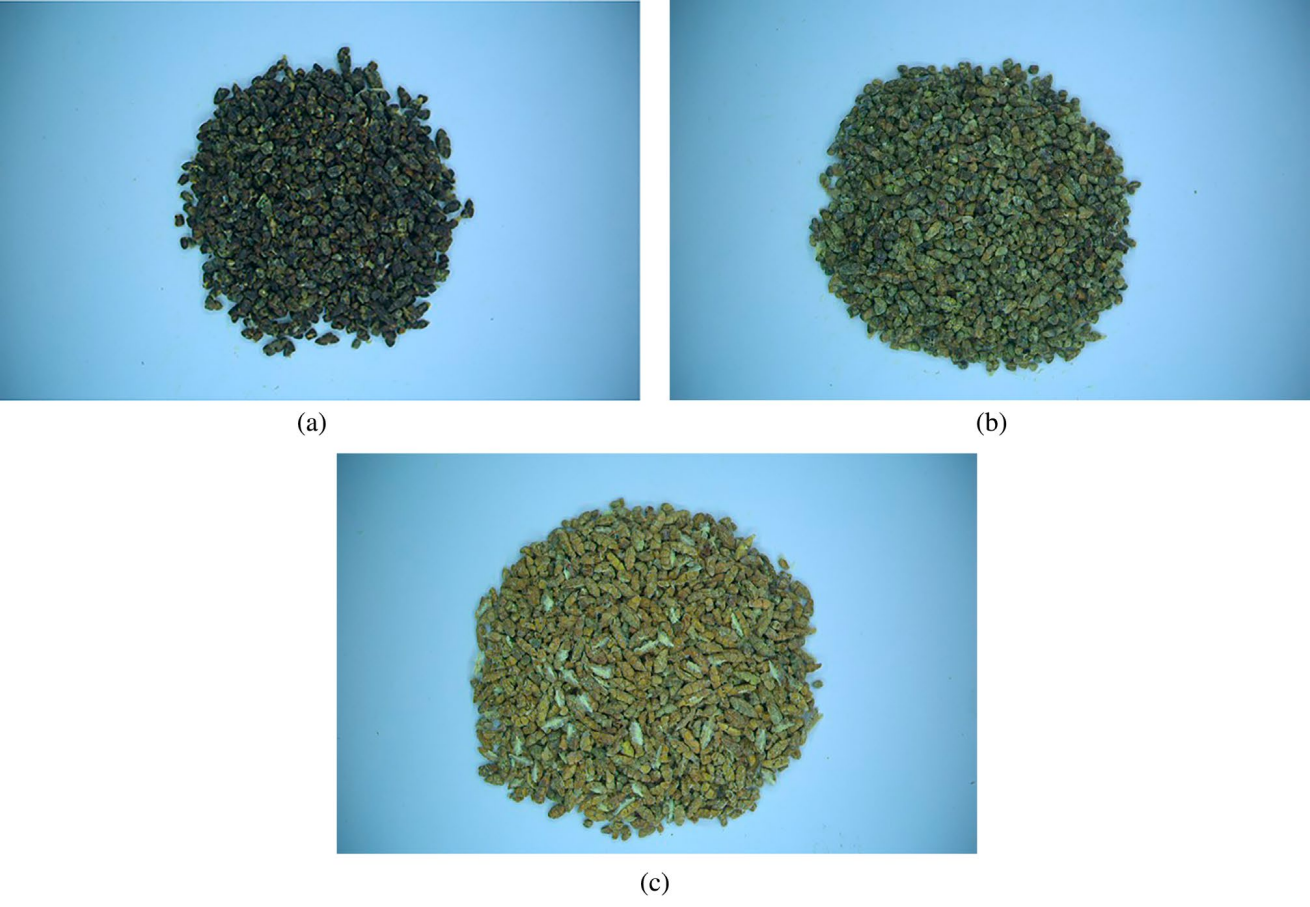
Images of different quality grades of cardamom seeds, (a) Grade 1, (b) Grade 2, and (c) Grade 3.

A CV system was used to acquire the color images of cardamom capsules and seeds. The system consisted of a wooden chamber with matte black inner walls, a lighting system composed of fluorescent lamps, a digital color camera connectable to a computer with a resolution of 2.3 megapixels (Basler acA1920‐40uc, Basler AG, Germany) equipped with a lens (Basler TS5014‐MP F1.4 f50 mm, Basler AG, Germany), and a user graphical interface (Basler's Pylon viewer software, Version 6.3.0.23157, Basler AG, Germany). The samples were placed on a matte white surface, and the camera was mounted above the samples at a vertical distance of 0.2 m. In each experiment, 20 g samples of cardamom capsules and seeds were placed under the camera. The color images with a size of 1200 × 1920 pixels were taken from each sample using the camera's software and user interface. The CV system used to acquire color images of cardamom capsules and seeds is shown in Figure 4. Samples of the images obtained from the different classes of cardamom capsules and seeds studied in this research are presented in Figures 5 and 6, respectively.

The color images of the cardamom samples were called in the image processing toolbox of MATLAB software (The Mathworks, R2021a) for processing and feature extraction. In order to reduce and simplify the image processing steps, first a 400 × 400‐pixel block was cropped from the center of the color images of the samples using the “*imcrop*” function. This block only included the image of the sample and did not include the background of the image. To extract color features, the blocks were converted from the RGB color space to the HSV and L*a*b color spaces. After color space conversion, the mean, standard deviation, skewness, and kurtosis values of red (*R*), green (*G*), blue (*B*), hue (*H*), saturation (*S*), intensity (*V*), lightness (*L*), and color components of the blocks were extracted (Sangwine and Horne 1998). The conversion from RGB color space to HSV and L*a*b color spaces was performed in MATLAB software using “*rgb2hsv*” and “*rgb2lab*” functions, respectively.

To extract texture features, the image blocks were first converted to grayscale images using the “*rgb2gray*” function, and then the gray level co‐occurrence matrix (GLCM) was created for the grayscale images. The components of the GLCM matrix represent the number of repetitions of pairs of pixels with specific grayscale values in a specific spatial direction in the grayscale image. Mathematically, the co‐occurrence matrix (*P*) for an image I of dimensions *m* × *n* in the direction (∆*x*, ∆*y*) is calculated using the following formula (Alzhanov and Nugumanova 2024):

(1) $$P_{∆x,∆y}\;\left(i,j\right)=\sum_{p=1}^{n}\sum_{q=1}^{m}\left\{\begin{array}{cc} 1 & \text{if}\;I\left(p,q\right)=i\;\text{and}\;I\left(p+∆x,q+∆y\right)=j\\ 0 & \text{Otherwise} \end{array}\right.$$

where $P_{∆x,∆y}\;\left(i,j\right)$ is the (*i*, *j*)‐th element of the co‐occurrence matrix *P*.

In this research, for each block, four GLCM matrices were calculated for four different directions (0°, 45°, 90°, and 135°) with a spatial distance of one pixel. The average of these four GLCM matrices was used to extract texture features (Bakhshipour and Zareiforoush 2020). In this study, 16 different texture features based on the GLCM matrix were calculated, which were entropy, energy, inertia, correlation, homogeneity, dissimilarity, sum of squares, sum of means, sum of variances, sum of entropies, variance of difference, entropy of difference, cluster shade, cluster prominence, inverse difference moment, and maximum probability. Descriptions and relationships of these texture features have been provided in previous studies (Bakhshipour et al. 2017; Haralick 1979; Park and Chen 2001). In total, 52 image features (36 color features and 16 texture features) were extracted from the image blocks and used for implementing the ML algorithms.

### 2.4 Feature Selection

Feature selection algorithms are used to reduce the dimensionality of the initial feature vector, leading to improved speed and accuracy in the classification process. Although with the advancement of computer science in hardware and software, the processing and computation speed in ML algorithms will not be significantly reduced even with a large number of elements in the initial feature vector, the main purpose of using feature selection algorithms is to remove some less important features. This significantly helps simplify the problem, reduce the complexity of the final classifier model, and increase its efficiency (Mollazade et al. 2013; Zareiforoush et al. 2016). One of the most common and well‐established feature selection methods in pattern recognition problems is the correlation‐based feature selection (CFS). CFS is a supervised feature filtering approach that can select an optimal subset of features from the original dataset based on a correlation function. This method ranks a subset of features based on the correlation coefficient index (Bakhshipour et al. 2020; Zareiforoush et al. 2016). The selectability of a specific feature depends on its ability to predict the target classes in a way that other features do not have such predictive ability. The evaluation function of the feature subset is calculated using the following equation (Russell and Norvig 2016):

(2) $$M_{s}=\frac{k\bar{r_{\mathrm{cf}}}}{\sqrt{k+k\left(k-1\right)r_{\mathrm{ff}}}}$$

where *M*_s_ represents the correlation between a feature set and the class, *k* is the number of components, $\bar{r_{\mathrm{cf}}}$ represents the average correlation between features and the class, and *r*_ff_ represents the average inter‐correlation between features.

Typically, there are three heuristic search strategies, including forward selection, backward elimination, and best‐first approach, in the CFS implementation process. In this study, the best‐first feature selection method was used to select the most effective features. In this method, the search for the best subset of features stops when there is no improvement in the correlation coefficient index in five consecutive generated subsets compared to the current best subset (Liu and Motoda 2007).

### 2.5 ML Algorithms

In pattern recognition problems, the goal is to assign some input objects to one of the given predefined categories called as classes. For this purpose, a classifier, which is an ML algorithm, is applied to find the relationship between the inputs (features) and the output (classes) and correctly assign inputs to their corresponding labels (Abe 2005). Different algorithms can be used to classify data based on a set of input features. In this study, three methods including SVM, Bayesian Network (BN), and DT were used for quality classification of cardamom. These algorithms are among the most commonly used classification methods for data analysis in e‐nose and CV applications (Anwar et al. 2023; Zhu et al. 2021).

SVMs, also known as kernel‐based methods, are widely used in pattern recognition problems. Learning an SVM classifier is to find a descriptive equation for a multidimensional surface that can effectively separate different classes in the feature space. By taking a training sample, SVM builds a hyperplane as the decision surface so that the distance of positive and negative examples from the hyperplane is maximized (Haykin 2009). One of the advantages of the SVM method is that, unlike classifiers such as genetic algorithms and artificial neural networks (ANNs), it provides the same optimal hyperplane parameter for distinguishing classes in any number of executions for a specific data set. For a specific kernel function that transforms the data from the input space to the feature space, parameters are obtained uniquely for an SVM model, for a given training set, while models based on genetic algorithms and ANNs are obtained differently each time as a result of the training process (Awad et al. 2015). In this study, different kernel functions including Polynomial Kernel, Normalized Polynomial Kernel, Pearson VII Function‐based Universal Kernel (PUK), and RBF Kernel were used to develop the SVM classifying models.

Classifications can be carried out using a probabilistic approach, with BNs being widely used as classifiers in this context. BNs provide a probabilistic graphical model that calculates the conditional dependence of a set of random variables based on Bayes' theorem (Liu et al. 2015). Considering the dependencies and conditional probabilities, BNs construct a directed acyclic graph, in which no loop or self‐connection is possible. In these graphs, nodes correspond to variables (continuous or discrete), and arcs represent the conditional probabilities based on causality relationships (Yang 2019). In this study, in order to select the best BN model for cardamom grading, different search algorithms including K2 search, genetic search, hill‐climber search, and simulated annealing search were investigated. These algorithms have been proven effective in the quality classification of agricultural products (Mollazade et al. 2012; Zareiforoush et al. 2016).

DTs are another widely used and effective supervised learning algorithms for classification tasks (Paul et al. 2018). The DT algorithm is essentially a hierarchical data structure that operates based on the divide‐and‐conquer strategy. This efficient non‐parametric approach can be used for both classification and regression purposes. The result of a DT algorithm is a tree‐like structure consisting of leaves, branches, and internal nodes. Each internal node in the tree structure represents a different pairwise comparison of a selected feature, while each branch represents the outcome of this comparison. Leaf nodes represent the final decision or prediction that is obtained after traversing the path from the root to the leaf, which is expressed as a classification rule (Alpaydin 2020; Russell and Norvig 2016). In this study, two DT algorithms of REP and J48, which are popular DT algorithms in supervised classification, were used for the quality classification of cardamom capsules and seeds. These algorithms have been successfully used in previous studies to grade quality indices of food and agricultural products (Bakhshipour et al. 2018; Liakos et al. 2018; Santana et al. 2024; Wang et al. 2022).

### 2.6 Data Fusion

Data fusion is a powerful technique that integrates data from multiple sources to achieve more accurate and reliable information than what could be obtained from any individual source alone (Durrant‐Whyte and Henderson 2016). The primary goal of data fusion is to create a comprehensive and predictive model of a system by leveraging data from multiple independent sensor systems (Gutiérrez et al. 2013). Data fusion is performed at three different levels, including signal level, feature level, and decision level (Weckenmann et al. 2009). In signal‐level data fusion, the input data is combined in its original form. At the feature level, as the name suggests, the features extracted from different sensors are combined. In decision‐level fusion, decisions (e.g., classification results) obtained from separate models are combined (Wang et al. 2015). In this study, the feature‐level method was used to fuse data obtained from e‐nose and CV systems.

### 2.7 Performance Evaluation of ML Models

The extracted data were randomly divided into two groups: 70% for the calibration (training) process and 30% for evaluating the classification models. In the calibration stage of the models, the 10‐fold cross‐validation method was used (Huang and Gu 2022). The best classifiers were chosen based on the highest accuracy and the lowest root mean squared error (RMSE) (Liu et al. 2018). All stages of data partitioning, feature selection, implementation, and evaluation of the ML algorithms were carried out in the WEKA software environment.

## 3 Results and Discussion

### 3.1 Cardamom Capsule Classification

#### 3.1.1 CV Method

To select the most relevant image features for the classifiers, the CFS algorithm was used. As can be seen in Table 2, among 52 color and texture features extracted from the images of cardamom capsule samples, the CFS method selected 8 features as optimal indicators to determine different quality grades of the product. Such a reduction in feature complexity simplifies and expedites the classification process. This advantage enables the development of an online cardamom capsule grading system. As observed, the majority of the selected features are color features. This finding aligns with the criteria defined in cardamom capsule and seed grading standards, where color is a primary and crucial factor in determining the product quality grade.

**TABLE 2:** Result of the CFS algorithm on image features of cardamom capsule samples.

Number of features	Selected features
8	R_ave, G_ave, G_kurt, B_std, H_ave, H_std, Maximum_probability, and Sum_entropy

The performance metrics of the classifiers evaluated in this study for classifying cardamom capsule samples using image color and texture features are presented in Table 3. It is observed that the highest classification accuracy on the calibration data was 96.67%, achieved by the BN method using the CFS‐based features. The RMSE value of this classifier on the calibration data was 0.1408. As mentioned in the previous section, the CFS algorithm selected only eight features from the cardamom capsule images as optimal features. The high performance achieved by the CFS‐BN model demonstrates the strong potential of combining CV and ML algorithms for developing a non‐destructive cardamom capsule grading system. The CFS‐BN algorithm was evaluated on a separate dataset and achieved an accuracy of 93.33%.

**TABLE 3:** Results of SVM, BN, and DT models for classification of cardamom capsule samples by the use of image features.

Classifier	Feature selection method	Accuracy	RMSE
SVM	—	63.33	0.4944
	CFS	93.33	0.2109
BN	—	93.33	0.1987
	CFS	96.67	0.1408
J48 DT	—	93.33	0.2214
	CFS	93.33	0.2108

The confusion matrices of the CFS‐BN model for the calibration and evaluation phases are presented in Table 4. This table indicates that only 1 out of 30 samples was misclassified in the calibration phase, and similarly, only 1 out of 15 samples was misclassified in the evaluation phase.

**TABLE 4:** Confusion matrix of the CFS‐BN model for classification of different degrees of cardamom capsules based on image features.

Class	Calibration			Evaluation		
	Grade 1	Grade 2	Grade 3	Grade 1	Grade 2	Grade 3
Grade 1	10	0	0	5	0	0
Grade 2	1	9	0	1	4	0
Grade 3	0	0	10	0	0	5

Successful applications of CV systems in previous research have also been reported in various fields related to agricultural products and the food industry. In the context of using CV systems for grading raisins, a study showed that by using image processing, it is possible to separate dark and light raisins from each other and the background with an accuracy of 92% (Khojastehnazhand and Ramezani 2020). Shrivastava and Pradhan (2021) investigated the classification of rice diseases using color features by examining 14 different color spaces. Four features were extracted from each color channel, resulting in a total of 172 features. They showed that the highest classification accuracy of 94.65% was achieved using the SVM classifier (Shrivastava and Pradhan 2021). Another study investigated the classification of six different medicinal herb species using a neural network classifier. Various feature extraction techniques, including morphological, color, and texture‐based methods, were employed. The extracted features from a dataset of 90 images were analyzed, and the overall classification accuracy of the BPNN classifier was found to be 98.88% (Saikia et al. 2022). The effectiveness of ML algorithms in the recognition and classification of medicinal herbs based on image‐extracted features, such as color and texture, has been demonstrated by another researcher (Kadiwal et al. 2023).

#### 3.1.2 E‐Nose Method

**FIGURE 7:**
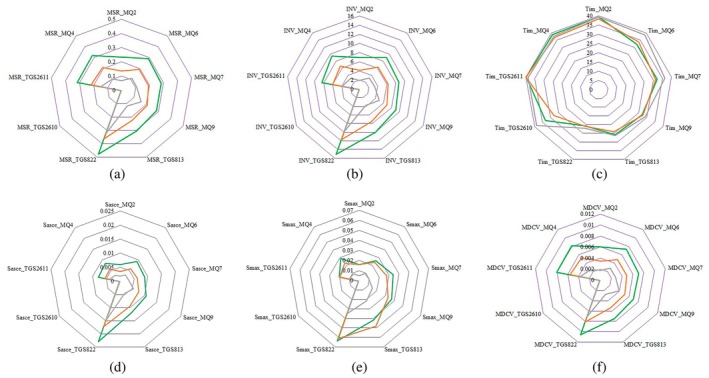
Radar diagrams of extracted features based on e‐nose from different quality grades of cardamom capsules: (a) MSR, (b) INV, (c) *T*_im_, (d) *S*_asce_, (e) *S*_max_, and (f) MDCV (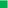 Grade 1, 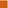 Grade 2, and 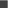 Grade 3).

Figure 7 presents the mean values of the features extracted from each of the nine sensors in the e‐nose system for different quality grades of cardamom capsules. In most of the features extracted from the sensor responses, an obvious difference between different quality grades of the samples is observed. This indicates that the information from the e‐nose sensors can be used to discriminate between different quality grades of cardamom capsules. According to Figure 7, the distinction between the quality classes of cardamom capsules in the graphs for some measures such as MSR and MDCV is more pronounced compared to other graphs, which shows the superiority of these indices for classification. However, it should be kept in mind that due to the high number of calculated e‐nose features, some of these features may have a high inter‐correlation or some features do not contain appropriate discriminating information for the product classification. In order to accurately determine which of the extracted features are more effective for the classification process, it is necessary to employ a feature selection algorithm. For this purpose, the CFS algorithm was applied to the e‐nose‐extracted data.

As can be seen in Table 5, by applying the CFS method to the 54 input variables from the e‐nose system, 23 features were selected as optimal features for classifying cardamom capsule samples. It was observed that the selected features were of different types and from different sensors. This indicates that the combination of sensors and the combination of calculated parameters is necessary for the proper quality classification of the cardamom capsule, and relying on one or a limited number of sensors may not produce satisfactory results.

**TABLE 5:** Selected features from the e‐nose method using the CFS algorithm for the classification of cardamom capsules.

Number of features	Selected features
23	MSR_MQ2, MSR_MQ6, MSR_MQ7, MSR_TGS813, MSR_TGS2611, MSR_MQ4, INV_MQ2, INV_MQ6, INV_MQ7, INV_MQ9, INV_TGS2611, INV_MQ4, Sasce_MQ2, Sasce_MQ6, Sasce_MQ7, Sasce_MQ4, Smax_MQ7, Smax_MQ4, MDCV_MQ2, MDCV_MQ6, MDCV_MQ7, MDCV_MQ9, and MDCV_MQ4

To perform cardamom capsule classification based on e‐nose data, the aroma‐based feature vectors were introduced to the modeling algorithms in two ways. First, all 54 features extracted from the e‐nose system were used to develop the models, and then the features selected by the CFS algorithm were used to train the classifying algorithms. Table 6 shows the performance metrics of the most accurate models for the quality classification of cardamom capsules using e‐nose data. In this case, the BN model, using both overall data and CFS‐based data, and the SVM model, using CFS‐based data, achieved the highest classification accuracy (97.67%) in the calibration stage. Given the lowest RMSE value (0.1126), the CFS‐BN model was found to be more favorable than the other developed algorithms. Evaluation of the CFS‐BN model with separate data showed that the model's classification accuracy in the evaluation stage was 93.33%, correctly classifying 14 out of 15 samples. The confusion matrix of the CFS‐BN model is presented in Table 7.

**TABLE 6:** Results of SVM, BN, and DT models for classification of cardamom capsule samples by the use of e‐nose features.

Classifier	Feature selection method	Accuracy	RMSE
SVM	—	90.00	0.2582
	CFS	96.67	0.1491
BN	—	96.67	0.1467
	CFS	96.67	0.1126
J48 DT	—	90.00	0.2578
	CFS	93.33	0.2108

**TABLE 7:** Confusion matrix of the CFS‐BN model for classification of different degrees of cardamom capsules based on e‐nose features.

Class	Calibration			Evaluation		
	Grade 1	Grade 2	Grade 3	Grade 1	Grade 2	Grade 3
Grade 1	10	0	0	5	0	0
Grade 2	0	10	0	1	4	0
Grade 3	0	1	9	0	0	5

Tohidi et al. (2019) investigated the applicability of an e‐nose system based on MOS sensors as a non‐destructive tool for geographical origin discrimination of three spices, namely, black pepper, cinnamon, and turmeric. Various ML methods were employed for the classification of these products. They concluded that the e‐nose system based on MOS sensors in combination with chemometrics can be an effective and useful tool for rapid and non‐destructive classification of black pepper, cinnamon, and turmeric in terms of geographical origin. In a study, it was demonstrated the application of a piecewise feature selection on the e‐nose data improves the classification rate for quality grading of black tea. The PCA and LDA methods were used for dimensionality reduction. The highest reported accuracy was 99.50%, which was achieved by the SVM classifier model (Kombo et al. 2024).

#### 3.1.3 Data Fusion of CV and E‐Nose

By applying the CFS method, 29 features were selected out of 106 general input variables from the combined CV and e‐nose data as optimal features for capsule classification (Table 8). It is observed that most of the selected optimal features by the CFS algorithm for the classification of cardamom capsules were obtained from the e‐nose method.

**TABLE 8:** Selected features from the fusion of CV and e‐nose data for cardamom capsule classification.

Number of features	Selected features
29	R_ave, R_skew, H_ave, Bs_ave, Sum_average, Sum_entropy, MSR_MQ2, MSR_MQ6, MSR_MQ7, MSR_TGS813, MSR_TGS2611, MSR_MQ4, INV_MQ2, INV_MQ6, INV_MQ7, INV_MQ9, INV_TGS2611, INV_MQ4, Sasce_MQ2, Sasce_MQ6, Sasce_MQ7, Sasce_MQ4, Smax_MQ7, Smax_MQ4, MDCV_MQ2, MDCV_MQ6, MDCV_MQ7, MDCV_MQ9, and MDCV_MQ4

Table 9 presents the performance indicators of the most accurate models for the qualitative classification of cardamom capsules through the fusion of e‐nose and CV data. In this condition, SVM and BN models achieved 100% accuracy in classifying cardamom capsules. In this case, the CFS‐BN model with the lowest RMSE value (0.1051) outperformed the other evaluated algorithms. Evaluation of this model on a separate dataset demonstrated that the data fusion of e‐nose and CV methods can differentiate the evaluation stage with 100% accuracy. The results of the performance evaluation of the classifiers based on the data fusion method compared to the separate use of the features obtained from the CV and e‐nose systems clearly demonstrate that the fusion of information from e‐nose sensors with CV system can significantly improve the detection capability and confidence in the quality grading of cardamom capsules.

**TABLE 9:** Performance metrics of SVM, BN, and DT classifiers for classification of cardamom capsule based on the fusion of e‐nose and CV data.

Classifier	Feature selection method	Accuracy	RMSE
SVM	—	93.33	0.2106
	CFS	100.00	0.1243
BN	—	96.67	0.1441
	CFS	100.00	0.1051
J48 DT	—	93.33	0.2108
	CFS	93.33	0.2106

In a study by Kiani, Minaei, and Ghasemi‐Varnamkhasti (2017), data fusion from CV and e‐nose methods using PCA and SVM algorithms resulted in 100% accuracy in saffron quality classification. The fusion of CV and e‐nose information has also led to more robust and improved prediction performance for postharvest quality evaluation of tomatoes (Huang et al. 2018) and spinaches (Huang et al. 2019).

### 3.2 Cardamom Seed Classification

#### 3.2.1 CV Method

As a result of applying the CFS algorithm, it was found that out of the 52 color and texture features extracted from the cardamom seed images, 27 features were selected as the optimal indicators for distinguishing different quality grades of the seeds (Table 10). It can be observed that the number of selected features for cardamom seed classification was significantly higher than that of cardamom capsules, which were only eight features (Table 2). In the case of cardamom seeds, most selected features were color‐based. Therefore, in the quality classification of cardamom seeds based on image processing methods, color indices offer more practical utility for use in cardamom grading and sorting systems as compared with the texture indices.

**TABLE 10:** Result of the CFS algorithm on image features of cardamom seed samples.

Number of features	Selected features
27	R_ave, R_std, R_skew, G_ave, G_skew, B_ave, B_std, H_ave, H_std, H_skew, H_kurt, S_ave, S_skew, V_ave, V_skew, Ls_ave, Ls_skew, Ls_kurt, As_ave, As_kurt, Bs_ave, Bs_skew, Bs_kurt, Correlation, Sum_of_sqaures, Sum_average, and Sum_variance

The performance specifications of the classification algorithms for separating different quality grades of cardamom seeds based on image features are presented in Table 11. In terms of classification accuracy, three classification models, including the BN model using all data, the CFS‐BN model, and the CFS‐SVM model resulted in the highest accuracy (96.67%) for cardamom seeds classification. Among these models, the CFS‐BN model was selected as the best classifier due to its lowest RMSE value (0.1220). The classification accuracy of the CFS‐BN model in the evaluation stage was found to be 93.33%. The confusion matrices of this model for classifying cardamom seeds in the calibration and evaluation stages were similar to the confusion matrices of the CFS‐BN model for classifying cardamom capsule samples using e‐nose features (Table 7).

**TABLE 11:** Performance characteristics of SVM, BN, and DT models for classification of cardamom seeds using image features.

Classifier	Feature selection method	Accuracy	RMSE
SVM	—	93.33	0.2106
	CFS	96.67	0.1251
BN	—	96.67	0.1376
	CFS	96.67	0.1220
J48 DT	—	90.00	0.2582
	CFS	93.33	0.2107

The possibility of quality grading of food grains using the combination of CV and ML algorithms has been also investigated by other researchers. In a study on classifying different varieties of corn kernels using image processing, it was reported that the classification accuracies of LDA and ANN classifiers were 70.6% and 75.6%, respectively (Rasekh et al. 2024). Macuácua et al. (2023) employed CV and ML models for the automatic classification of dried bean seeds. They utilized RF DT, SVM, and K‐nearest neighbors (KNN) algorithms. Their findings revealed that data mining processes like PCA can enhance the classification results. The KNN classifier achieved an accuracy of 95% (Macuácua et al. 2023). Koklu and Ozkan (2020) aimed to classify dry beans using CV and ML techniques. They evaluated MLP, SVM, KNN, and DT classifiers using 10‐fold cross‐validation and reported that the SVM model achieved the best classification performance with an accuracy of 93.13%. According to these reports, the performance of the superior classifier in this section of our study is at an acceptable level.

#### 3.2.2 E‐Nose Method

**FIGURE 8:**
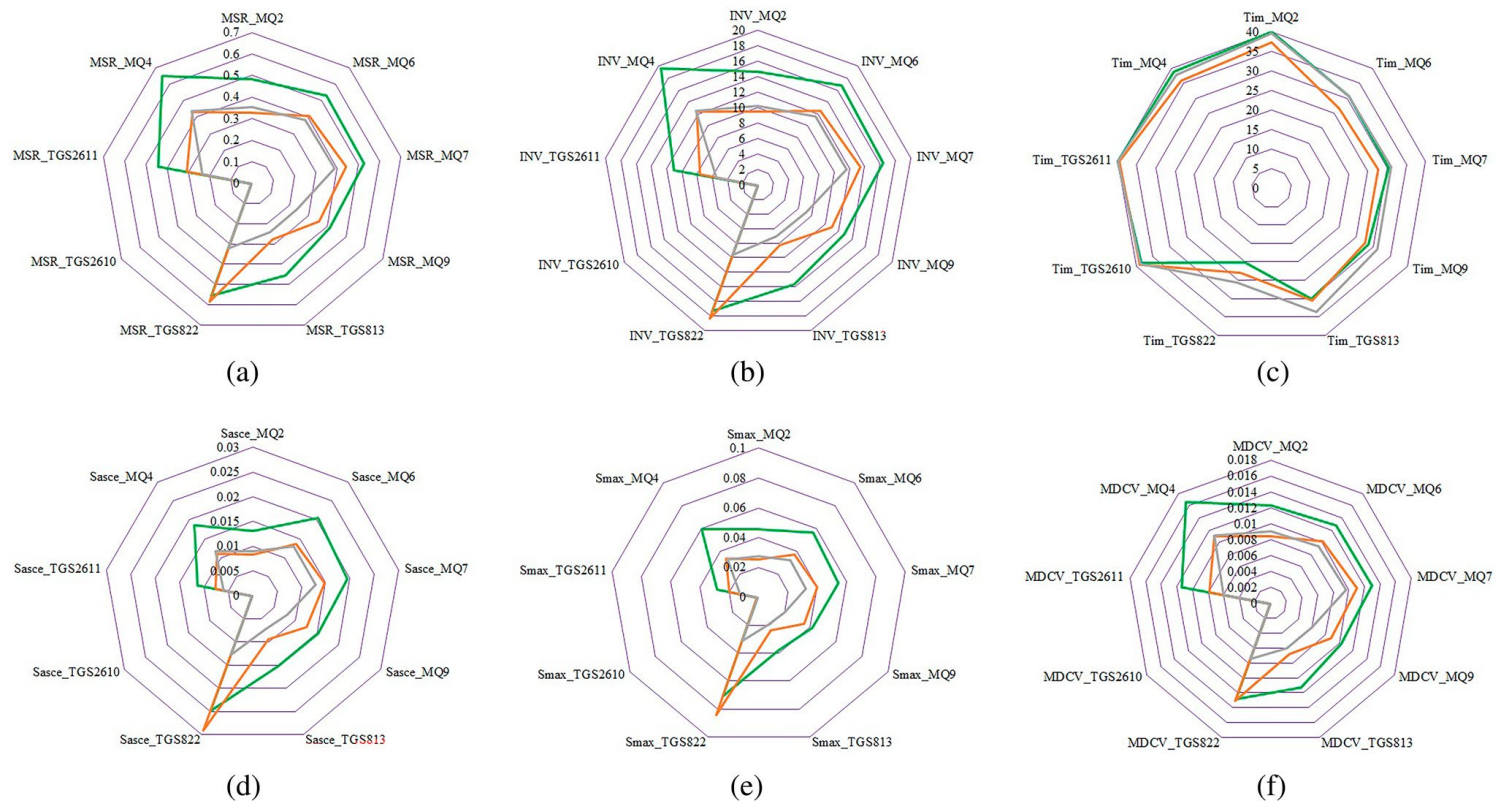
Radar diagrams of extracted features based on e‐nose from different quality grades of cardamom seeds: (a) MSR, (b) INV, (c) *T*_im_, (d) *S*_asce_, (e) *S*_max_, and (f) MDCV (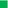 Grade 1, 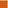 Grade 2, and 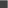
Grade 3).

Figure 8 shows the mean values of the features extracted from each of the nine sensors in the e‐nose system when exposed to the aroma of different qualitative grades of cardamom. In the MSR graphs, it can be observed that for most sensors, the samples of cardamom Grade 1 had the highest MSR values. This pattern is also observed in other features such as the area under the curve (INV feature) and the slope of the ascending part of the curve (*S*_asce_). This indicates that the aroma intensity in higher‐grade cardamom seeds was greater than that in seeds with lower‐quality grades. Consequently, the intensity of the aroma emitted by cardamom is an important indicator of their quality during the grading process. The results presented in Figure 8 show that the indices recorded by the gas sensors of the e‐nose system were the highest, in order, when exposed to the cardamom samples of Grade 1, 2, and 3. According to previous research, two compounds present in cardamom essential oil, 1.8‐cineole and α‐terpinyl acetate, are the main contributors to the aroma of cardamom seeds (Ashokkumar et al. 2021). The higher sensitivity of the e‐nose system's sensors to Grade 1 cardamom seeds compared to Grade 2 and Grade 3 seeds is likely due to the presence of higher amounts of the aromatic compounds 1.8‐cineole and α‐terpinyl acetate in Grade 1, which consists of black and oily seeds. Given that the e‐nose sensors detected significant differences in aroma intensity among Grade 1, 2, and 3 cardamom samples, this highlights the capabilities of intelligent systems based on e‐nose and ML methods for quality control and grading of cardamom in the commercial market.

Table 12 shows that by applying the CFS method to the 54 input variables from the e‐nose system, six features were selected as optimal features for classifying cardamom seed samples.

**TABLE 12:** Result of the CFS algorithm on e‐nose features for cardamom seeds classification.

Number of features	Selected features
6	MSR_TGS822, MSR_TGS2610, MSR_MQ4, Tim_MQ20, Smax_TGS822, Smax_TGS2611

Compared to the selected features of the e‐nose method for cardamom capsule classification, it can be seen that the number of final features selected for cardamom seed classification is significantly lower. This could be because the compounds responsible for the aroma of cardamom are mainly present in its seeds. As a result, stronger correlation coefficients have been established between the features extracted from e‐nose sensors and cardamom seed classes. Therefore, the classification process can be performed with a smaller number of these significant features. This finding increases the hope of having an intelligent system based on the e‐nose method for quick and accurate grading of cardamom seeds on a commercial scale.

Table 13 shows the accuracy and RMSE values of the developed models for the quality classification of cardamom seeds using e‐nose data. In this case, the DT and BN models, using both the entire feature set and the features selected by the CFS method, achieved the highest classification accuracy (93.33%) in the calibration stage. The RSME value of the CFS‐DT model (0.2093) was the lowest compared to the other developed algorithms, making it more favorable. The CFS‐BN yielded an RMSE value of 0.2102, demonstrating satisfactory performance. In the model evaluation stage, the CFS‐DT model achieved a 93.33% accuracy in classifying cardamom seeds, with only one misclassified sample. The confusion matrices for this model for classifying cardamom seeds in the calibration and evaluation phases are shown in Table 14. A previous study was conducted to identify and classify five different cumin varieties using an e‐nose system based on eight MOS sensors and chemometrics. It was reported that the classification accuracy achieved using the SVM model was equal to 97.92% (Ghasemi‐Varnamkhasti, Tohidi, et al. 2018b). Other studies have also reported successful applications of e‐nose data combined with ML algorithms for food quality assessment. These include a 97.7% accuracy of an ANN in mint quality assessment (Kiani et al. 2023), an 84%–97% linear correlation in rice cultivars classification (Han et al. 2023), and a 98.8% accuracy of an ANN for classifying coffee bean types based on roasting time and degree (Astuti et al. 2024).

**TABLE 13:** Performance metrics of SVM, BN, and DT models for the classification of cardamom seeds using e‐nose‐based features.

Classifier	Feature selection method	Accuracy	RMSE
SVM	—	86.67	0.2559
	CFS	90.00	0.2392
BN	—	93.33	0.2113
	CFS	93.33	0.2102
J48 DT	—	93.33	0.2121
	CFS	93.33	0.2093

**TABLE 14:** Confusion matrices of the CFS‐DT model for the classification of different degrees of cardamom seeds based on e‐nose features.

Class	Calibration			Evaluation		
	Grade 1	Grade 2	Grade 3	Grade 1	Grade 2	Grade 3
Grade 1	10	0	0	5	0	0
Grade 2	0	10	1	1	4	0
Grade 3	0	1	9	0	0	5

#### 3.2.3 Data Fusion of CV and E‐Nose

Table 15 shows that by applying the CFS method to the total 106 input features from the combined data of CV and e‐nose, 30 features were selected as optimal features for classifying cardamom samples. It is observed that most of the optimal features selected by the CFS method for cardamom seed classification are the image‐based data.

**TABLE 15:** Selected features from the fusion of CV and e‐nose data for cardamom seeds classification.

Number of features	Selected features
30	R_ave, R_std, R_skew, G_ave, G_skew, B_ave, B_std, H_ave, H_std, H_skew, H_kurt, S_ave, S_skew, V_ave, V_skew, Lss_ave, Ls_skew, Ls_kurt, As_ave, As_kurt, Bs_ave, Bs_skew, Bs_kurt, Correlation, Sum_of_sqaures, Sum_average, Sum_variance, MSR_TGS813, Tim_TGS822, and Sasce_TGS813

Table 16 presents the performance characteristics of the most accurate models for quality classification of cardamom seeds based on the data fusion of e‐nose and CV methods. The results demonstrate that all models exhibited appropriate performance, indicating the high capability of aroma‐extracted features in discriminating different quality classes of cardamom seeds. In this study, the CFS‐BN model was selected as the best classifier for cardamom seeds based on the combination of e‐nose and CV data, achieving 100% accuracy and an RMSE of 0.0993 in the calibration phase. The accuracy of this model was also 100% in the model evaluation stage. The results demonstrate that the fusion of data obtained from CV and e‐nose methods, in combination with ML algorithms, leads to improved classification accuracy of cardamom seeds compared to using either method alone.

**TABLE 16:** Performance metrics of SVM, BN, and DT classifiers for classification of cardamom seeds based on the fusion of e‐nose and CV data.

Classifier	Feature selection method	Accuracy	RMSE
SVM	—	96.67	0.1227
	CFS	96.67	0.1160
BN	—	96.67	0.1104
	CFS	100.00	0.0993
J48 DT	—	93.33	0.2076
	CFS	96.67	0.1320

In a study on rapid quality assessment of black tea using NIRS, e‐nose, and data fusion, it was found that the integration of NIRS and e‐nose data effectively classifies different quality grades of tea based on their taste and aroma, with accuracies of 98.13% for SVM, 96.63% for KNN, and 97.75% for ANN (Xia et al. 2024). Other researchers have reported 100% accuracy in classifying the quality of tea using a combination of e‐nose and CV data fusion and the SVM algorithm (Xu et al. 2019). Wang et al. (2023) reported a classification accuracy of 99.71% for the origin of black pepper using data fusion from three methods: electronic tongue (E‐tongue), e‐nose, and electronic eye (e‐eye), combined with convolutional neural network (CNN) models.

## 4 Conclusion

In this research, the capabilities of CV and e‐nose systems in combination with ML algorithms were studied for the quality grading of cardamom capsules and seeds. Using the mentioned systems, various features were extracted from the images and odor‐related signals of the samples. The CFS‐BN algorithm was able to classify cardamom samples in different states with very high accuracy (over 93.33%) using e‐nose data and separate CV data.

The results of the classifiers' performance evaluation based on the data fusion method, compared to the separate use of features obtained from CV and e‐nose systems, clearly showed that combining e‐nose sensors information with CV can significantly improve the detection capability and confidence in the quality grading of cardamom capsules and seeds. The accuracy of the combined system with the application of the CFS‐BN model in cardamom product grading was 100% in both the calibration and evaluation stages. Compared to the limitations of e‐nose systems, such as the time required for the sample to stop in the sample chamber, CV systems can perform online data acquisition. In conclusion, CV systems can be proposed for standardized, online, and non‐destructive grading of cardamom capsules and seeds with high accuracy and confidence. Based on the results of this research, it can be concluded that by using the physical characteristics and features extracted from the odor of cardamom products, and by employing ML algorithms, the quality grading of different classes of cardamom capsules and seeds can be performed with very high accuracy and reliability. This study provides valuable insights for the development of a fast, accurate, and non‐destructive system based on the fusion of CV and e‐nose data for quality the grading of cardamom products.

## Author Contributions

**Ehsan Godini:** conceptualization (supporting), data curation (supporting), formal analysis (supporting), investigation (supporting), methodology (supporting), writing – original draft (supporting), writing – review and editing (supporting). **Hemad Zareiforoush:** conceptualization (lead), data curation (equal), formal analysis (equal), funding acquisition (lead), investigation (lead), methodology (equal), project administration (equal), software (equal), supervision (equal), validation (equal), visualization (equal), writing – original draft (equal), writing – review and editing (equal). **Adel Bakhshipour:** conceptualization (equal), data curation (equal), formal analysis (equal), funding acquisition (equal), investigation (equal), methodology (equal), project administration (equal), software (equal), supervision (equal), validation (equal), visualization (equal), writing – original draft (equal), writing – review and editing (equal). **Zahra Lorigooini:** conceptualization (supporting), formal analysis (supporting), funding acquisition (supporting), investigation (supporting), methodology (supporting). **Sayed Hossain Payman:** data curation (supporting), funding acquisition (supporting), resources (equal).

## Conflicts of Interest

The authors declare no conflicts of interest.

## Data Availability

The authors have nothing to report.
